# The application framework of big data technology during the COVID-19 pandemic in China

**DOI:** 10.1017/S0950268822000577

**Published:** 2022-03-29

**Authors:** Wenyu Chen, Ming Yao, Liang Dong, Pingyang Shao, Ye Zhang, Binjie Fu

**Affiliations:** 1 Department of Respiration, Affiliated Hospital of Jiaxing University, Jiaxing, China; 2 Anesthesia and Pain Medicine Center, Affiliated Hospital of Jiaxing University, Jiaxing, China; 3 Department of Information, Affiliated Hospital of Jiaxing University, Jiaxing, Zhejiang 31400, China; 4 Department of General Practice, Affiliated Hospital of Jiaxing University, Jiaxing, Zhejiang 31400, China

**Keywords:** Big data, COVID-19, epidemic prevention and control

## Abstract

Big data has been reported widely to facilitate epidemic prevention and control in health care during the coronavirus disease 2019 (COVID-19) pandemic. However, there is still a lack of practical experience in applying it to hospital prevention and control. This study is devoted to the practical experience of design and implementation as well as the preliminary results of an innovative big data-driven COVID-19 risk personnel screening management system in a hospital. Our screening system integrates data sources in four dimensions, which includes Health Quick Response (QR) code, abroad travelling history, transportation close contact personnel and key surveillance personnel. Its screening targets cover all patients, care partner and staff who come to the hospital. As of November 2021, nearly 690 000 people and 5.79 million person-time had used automated COVID-19 risk screening and monitoring. A total of 10 376 person-time (0.18%) with abnormal QR code were identified, 242 person-time with abroad travelling history were identified, 925 person-time were marked based on the data of key surveillance personnel, no transportation history personnel been reported and no COVID-19 nosocomial infection occurred in the hospital. Through the application of this system, the hospital's expenditure on manpower and material resources for epidemic prevention and control has also been significantly reduced. Collectively, this study has proved to be an effective and efficient model for the use of digital health technology in response to the COVID-19 pandemic. Based on the data from multiple sources, this system has an irreplaceable role in identifying close contacts or suspicious person, and can significantly reduce the social burden caused by COVID-19, especially the human resources and economic costs of hospital prevention and control. It may provide guidance for clinical epidemic prevention and control in hospitals, as well as for future public health emergencies.

## Introduction

Coronavirus disease 2019 (COVID-19) is highly infectious and spreads rapidly [1, 2], which was listed as a Class B infectious disease and management in accordance with Class A infectious diseases on 20 January 2020 in China. The outbreak of COVID-19 has posed a major challenge to China and the international response to public health emergencies [3]. Although China has effectively controlled the local epidemic in the early stage of the epidemic, there still is the risk of sporadic outbreaks, local outbreaks and foreign imports due to the significant population mobility.

Hospitals have been facing significant challenges not only because of the epidemic itself but also the difficulties in providing routine health care services, such as the care for chronic conditions and emergency visits [1, 4, 5] during the pandemic. Recently, several nosocomial infections of COVID-19 have been reported [1]. The large population mobility made the hospital be at a high risk of COVID-19 cross-infection [4, 6]. Furthermore, there is also a type of COVID-19 patients that do not show any symptoms, which usually is both challenging for themselves to realise their infection and for others to reduce the infection risk from them. Asymptomatic COVID-19 patients have become the focus and the difficulties of the current epidemic prevention and control work in hospitals. How to control the source of infection, block transmission and protect the vulnerable population in the hospital become a priority. Contact tracing and prehospital assessment could be essential. Strict pre-examination and triage measures, including epidemiological survey and temperature measurement, need to be taken before patients entering the hospital [5].

The prehospital epidemiological screening process in most hospitals often relies on traditional manual methods, such as oral and written surveys, which greatly depends on clinical experience. The manual operation is inefficient, of low accuracy, and may also increase the risk for cross-infection due to patients gathering at the hospital gates for a long time. Therefore, it is critical to developing an effective and efficient system to identify the potential patients with the risk of infecting COVID-19 and separate them from other patients and medical staff, thus minimising the risk of further outbreaks and ensure the least burden to the medical resources.

Big data has been reported widely to facilitate epidemic prevention and control in health care. It plays an important part in detecting cases, early warning and monitoring, identifying clustered epidemic situations accurately and quickly [7], Big data analysis provide technical support for epidemic screening such as personal information collection, trajectory query, transport networks and human temperature measurement [8, 9]. Big data technologies displayed in the form of epidemic maps, big data platforms, mobile application devices, etc. have been widely used [10, 11]. A number of countries around the world, including China, Singapore, France, Germany, Italy, Denmark and Australia have launched mobile phones and associated apps for contact tracing and tracking isolations/quarantines, such as ‘Health QR Code’, ‘close contact measuring instrument’ and ‘Trace Together’, etc. [12, 13]. These digital tools are all based on smartphones and use big data technology and complex algorithms to intelligently determine whether the user is a confirmed case or a potential source of infection [14, 15]. It may significantly improve the efficiency of epidemic management. However, there is still a lack of practical experience in applying big data technology to hospital prevention and control of major public health incidents, such as COVID-19. In response, in this article, we introduce the practical experience of design and implementation as well as the preliminary results of an innovative big data-driven COVID-19 risk personnel screening management system in order to provide more guidance for clinical epidemic prevention and control in hospitals.

## Methods

### COVID-19 screening system framework

As the only designated hospital for COVID-19 patients in the city, our hospital undertakes the task of treating infected patients. During the COVID-19 pandemic, our hospital has been authorised to access the data from the Health Commission of Zhejiang Province, which calls for the interface from the health department, the centre of disease control, and other non-health departments, to establish a big data-driven COVID-19 risk personnel screening system. The past two-week epidemiological data were collected from a wide range of sources and were presented in four forms, including (1) Health Quick Response (QR) code (2) abord travelling history, (3) transportation close contact personnel and (4) key surveillance personnel. Data integration and intelligent analysis through the screening system reading ID card numbers can be used to distinguish whether an individual is a COVID-19 risk person or not.

Health QR code is a big data technology application developed by China in the early stage of the epidemic, which is one of the criteria to assess if a patient is at high risk of COVID-19 [16]. It is classified in red, yellow and green colour levels [9] based on the data including name, ID number, mobile phone number, body temperature, cities, and travel data of the past 14 days; and whether they have the following symptoms: fever, cough and fatigue.

Red code including the following cases: confirmed patients, suspected patients, asymptomatic patients, persons who had close contact with confirmed patients, and persons under centralised isolation or medical observation at home. Yellow code including the following cases: persons with fever symptoms, fellow travellers who have no close contact with confirmed cases, persons who left a medium or high-risk epidemic area in the past 14 days, or other personnel subject to ‘yellow code’ management. Green code represents people other than the red and yellow code.

Although a health QR code can be used to display people's travel records and health status information, due to health QR code is based on the information provided by individuals, there is a risk of personal concealment and inaccurate data. Our screening system also utilised data from other nonself-reported sources to assess the risk of COVID-19 comprehensively. For instance, the travel and residence history information from the Ministry of Transport, Civil Aviation Administration, and Railway Corporation was used for contact tracing the high-risk population [10]. The discriminant interface of the key group is added to realise the rapid identification of key surveillance personnel in the country.

### Risk-rating model

This screening system mainly provides early warning to people at risk of COVID-19, so it has the characteristics of an ‘early warning system’. People who have a yellow or red health QR code will be identified as high-risk populations by our screening system. Similar to the yellow or red health QR codes, those who travelled abroad in the past 14 days will also be identified as high-risk populations by our screening system. People who travelled by train, plane, bus, or ship with the confirmed or suspected patients of COVID-19 within 14 days will be identified as high-risk populations as well. People who are COVID-19 confirmed or suspected patients, or their close contacts can be identified as high-risk populations. People who have been to epidemic or medium to high-risk areas, or in quarantine will be automatically identified and alerted.

### Screened population

#### Screening of online patients

To reduce the time spent in the hospital, the Internet Hospital [6] was first recommended to the people as a prehospital screening. The online fever clinic and specialist consultation service in our Internet hospital were first used to screen the people who plan to go to the hospital and for people self-screening. For each patient who is having an appointment with a doctor through online, before the patient comes to the hospital, we will also use the system to determine the risk of the patient from the patient's ID. Once a patient with a high risk of COVID-19 was identified by our Internet hospital or appointment registration channel, the patient would be recommended to the fever clinic first and cannot go to other departments of the hospital, which could minimise the of nosocomial infection from the source and avoid the time waste for the patients as well.

#### Screening of outpatients and visitors

Patients admitted through outpatient service should be screened at the hospital entrance, along with the patients who fail to complete the screening process online. Visitors are not recommended to accompany patients during special periods, considering that it is impossible to entirely forbid the visitor, our screening system also developed a procedure to cover both patients and their visitors.

#### Screening of inpatients and visitors

Face recognition information of inpatients and their visitors was recorded by inpatients and their visitors on the day of hospitalisation. They will be only authorised to access a certain area in the hospital. The information assessing COVID-19 risk could be obtained through the face recognition access control system before entering the authorised area. People who were not registered in the ward or who have a risk factor will not be allowed to enter the area, effectively protecting the medical care staff and patients.

#### Screening of hospital staff

Similar to the inpatients and visitors face recognition system, the hospital staff will also be screened by the employee access control system before they enter the working area, which synchronise with our screening system. When an employee is a risk person, the employee access control QR code will be synchronised into the same colour either in red or yellow as his health QR code.

### Offline verification and disposal

**Fig. 1.**
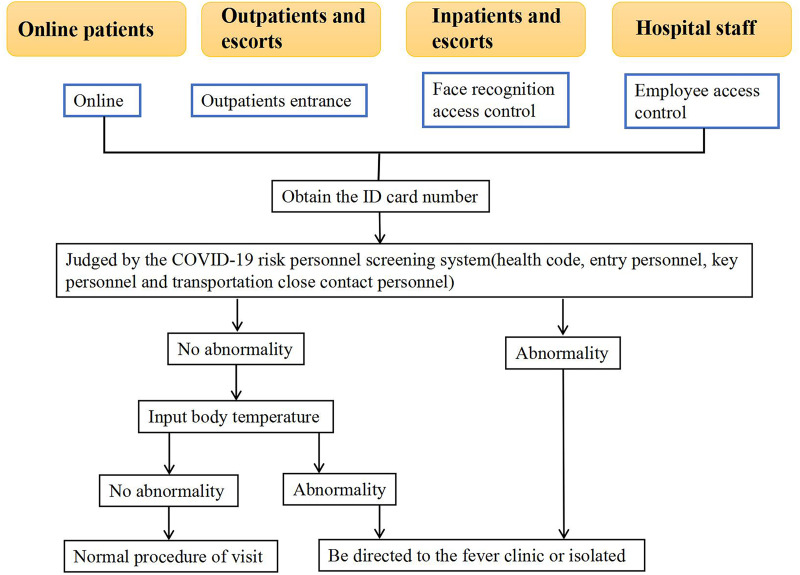
Hospital prevention and control management flow chart based on big data technology.

It takes people with high risk above assessed by the screening system as the key observation objects and further assessed by the health professionals in the fever clinic (such as isolation observation, nucleic acid testing), etc. Therefore, the whole hospital was monitored by the screening system effectively and require the least human efforts. The framework of this screening system is presented in Figure 1.

## Results

As of November 2021, nearly 690 000 people and 5.79 million person-time had used automated COVID-19 risk screening and monitoring. A total of 10 376 person-time (0.18%) with abnormal QR code were identified, including 2933 people with red health QR codes, 7443 people with yellow health QR codes. Besides, 242 people with abroad travelling history were identified through the abroad travelling history interface, 925 people were marked based on the data of key surveillance personnel from the interface of the Health Commission of Zhejiang Province, and there were no transportation close contact personnel been reported through the transportation history interface. For patients at risk to the fever clinic, after further assessment by the verification of artificial epidemiology, the coincidence rate of big data prediction is almost 100%. None of the people with a high risk for COVID-19 were tested positive. The COVID-19 caused a minimal level of effects on routine health care of the local hospitals.

**Fig. 2.**
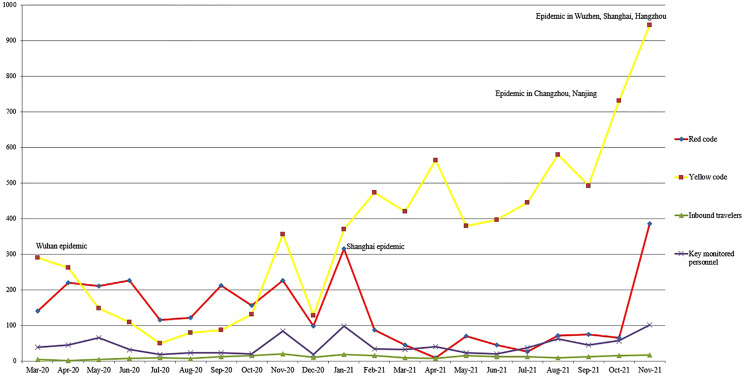
Trend chart of the number of high-risk people monitored by this screening system from March 2020 to November 2021.

The endemic situation in this city is stable and controllable, and there are no new local cases. However, there were several outbreaks in the surrounding areas of this city in January, October and November 2021. During this period of time, the number of high-risk persons for various reasons has increased with the development of the domestic epidemic, as shown in Figure 2. This shows the sensitivity of the early warning system to a certain extent.

In addition, through the use of this early warning system, the hospital's monthly expenditure on manpower, materials, etc. for prevention and control has been significantly reduced, and the psychological pressure of patients, their families, and volunteer workers on the epidemic seems to have been significantly reduced. See Table 1 for details. This shows that the precise prevention and control of the epidemic based on big data can reduce the burden on society.

**Table 1.** The difference between the hospital's average daily investment in labour expenses and material expenditures before and after the system is used

Burden	Before use	After use
Daily manpower (person)		
Entrance of outpatient Department building	16	4
Fever clinic entrance	4	1
Entrance of inpatient Department building	8	2
Entrance of each inpatient Department ward	35	0
Average screening time (minutes)	5	0.05
Daily average PPE used (sets)	63	7
Public acceptance	Long queue up time, poor experience	No crowds, good experience

## Discussion

Although China has effectively controlled the epidemic in the early stage, the epidemic situation in the world is still very serious. As early as 2006, Tatem A. J. *et al*. [9] published research on the global transportation network and the spread of infectious diseases. They believe that the continuous growth of modern transportation and the characteristics of high speed and complex networks provide a powerful force for the spread of diseases. Sokadjo Y. M. *et al*. [17] based on global passenger air traffic data found that the greater air traffic, the higher the number of COVID-19 infections. Zhao *et al*. [18] based on the travel data, the correlation between population mobility, the number of cases detected in different places and the prevalence rate of source infection was explored. However, in the context of today's world economic integration, the long-term disruption of traffic with a region/country (particularly economically developed regions/countries) due to the spread of disease is a difficult choice. This makes researchers find that using real-time data of population activities seems to be an efficient and accurate prevention and control method for COVID-19. Previous studies on the application of big data technology mainly focused on the early warning and monitoring of infectious diseases such as Ebola, dengue fever and avian influenza in the past 20 years [19]. With the outbreak of COVID-19, big data technology has been widely applied in epidemic prediction and control [7, 20, 21]. According to the experience of many countries such as China and Korea, health authorities have been authorised to collect personal data for public health purposes rather than for other purposes [7, 22, 23]. Chinese government proposed that China would further use big data to accurately prevent and control the spread of COVID-19, and put forward a joint anti-epidemic prevention and control mechanism. It requires that the key epidemiological information should be provided to the specific department by all related companies, institutions and individuals, and technically shared smoothly between different government departments [5, 24].

In the battle against the outbreak integration of big data, such as transportation data and location-based services data, is used to model viral activity and provide a guide for health care policy maker [17, 18, 25]. Digital contact tracing via smartphone apps was established as a new public-health intervention in many countries [26]. China, Singapore and South Korea have applied mobile technologies with some measure of success [10, 27]. However, most application scenarios often rely on the use of smartphones, and the data source is relatively unitary. Furthermore, little information has been reported on the application of big data technology in the hospital prevention and control of major public health events. It is difficult to identify patients and verify epidemiological information in the traditional hospital screening process, which needs a lot of human resources and resources. This article introduced the practical experience of designing and implementing a risk screening system in response to the COVID-19 to provide more guidance for clinical epidemic prevention and control in hospitals. Preliminary data showed the COVID-19 risk screening developed and used in our hospital had good acceptability among the public and sustainably reduced the burden on routine care during the pandemic.

Through literature review, only Chinese Taipei introduced a case of epidemic prevention and control related to big data technology for hospital outpatient appointments. They screened the contact history and travel history of outpatient patients, with few data sources and small population coverage. Compared with our system, there are several advantages in our hospital prevention and control management based on big data technology. First, The most obvious difference is the multidimensional data source, based on the data from multiple sources of multiple dimensions, the accuracy and screening efficiency of our screening system has been improved compared with most other systems being used by other institutions based on the data from a single source. It is in a leading position in data acquisition technology for entry personnel and key monitoring personnel. Second, for the people who cannot use the health QR code, such as the elderly population without a smartphone, the use of this system is unlimited. Their risk level of COVID-19 can be assessed through the ID card number, other data interface information and Epidemiological questionnaire but not the health code in the phone. Third, the screening scope is hospital-wide, covering all the hospital personnel, not just outpatients and there is no omission. What's more, it can quickly obtain the information of high-risk populations. The whole process needs three seconds only, thus, it can avoid cross-infection in the hospital caused by people gathering together. This screening system mainly provides early warning to people at risk of COVID-19, so it has the characteristics of an ‘early warning system’. It has demonstrated irreplaceable advantages in saving resources. Dynamic and continuous monitoring and early warning can estimate the number of risk personnel according to the curve fluctuation trend, so as to adjust the human and material resources for prevention and control. Low cost is beneficial to long-term prevention and control work. Furthermore, it has broken the information barrier between various departments through big data sharing and achieved whole population coverage and whole field joint defence. Finally, the prehospital screening online system bought time to adopt effective control measures by the hospital before the patients arrived at the hospital and saved time.

Although no nosocomial infection occurred in the hospital, there was no positive COVID-19 case that screened by the system either; thus, the sensitivity and specificity of our screening cannot be tested and proved effectively. We consider that the failure of the system to detect COVID-19-positive patients may be related to the following reasons: (1). The epidemic situation in the area where our hospital is located is stable, and there are no new local cases; when there is a local epidemic outbreak in the surrounding area, it can be seen from the curve trend chart that the screening rate of high-risk personnel in this system is positively correlated with the development of the epidemic. To a certain extent, this also reflects the sensitivity of this system in epidemic prevention and control. (2). Through community grid management in China, once the information that a person needs to be isolated is found, the CDC or the community will contact that personnel and isolate that personnel as soon as possible. The significance of this system is to supplement the search for COVID-19 positive patients who slip through the net. More importantly, it improves the prevention and control efficiency and reduces the human and material costs of hospital prevention and control. This advantage is obvious in the results of this paper.

In addition, during more than one year of use, our staff also found some limitations of this system. First, delay in data collection is common in big data technology. For example, the update of health code status is often delayed by 3 to 4 h, which will lead to the failure of early detection and early warning. Second, low efficiency of data collection, difficulty in guaranteeing data quality and low efficiency of data use are often existing. Additionally, data sharing remains inadequate among different medical institutions, departments and regions. For example, some regions cannot recognise the health QR codes generated by other regions partly due to inadequate infrastructure for urban data collection, lack of community data resources, and lack of expertise in big data management [24].

Therefore, although big data technology has developed, there are still challenges. Data sharing across departments and regions should be effectively integrated and better applied to epidemic management. A dynamic information sharing system and an interconnected health care joint health defence system should be established. Meanwhile, there is still a lack of clear legal basis and emergency plan to protect personal privacy and data security.

## Conclusion

Despised the limitations above, However, this study established a big data-driven screening system for people at risk of COVID-19. This screening system mainly provides early warning to people at risk of COVID-19, so it has the characteristics of an ‘early warning system’. It takes people with abnormal system prompts as the key observation objects and further screens them (such as isolation observation, nucleic acid testing), etc. In this way, the observation population is narrowed, realised the precise prevention and control of COVID-19, thereupon then reducing the social burden caused by COVID-19, thereby improving the efficiency of the hospital's prevention of COVID-19, and providing more guidance for the hospital's clinical epidemic prevention and control. Our practice experience has proved to be an effective and efficient model for the use of digital health technology in response to the COVID-19 pandemic, which may provide guidance for clinical epidemic prevention and control for now and future to achieve a great leap to deal with major public health events. Further research will be needed for data safety and data sharing in order to improve the effectiveness and efficiency of the infectious disease screening system.

## Data Availability

The data that support the findings of this study are available from the corresponding author upon reasonable request.
